# The glycemic, insulinemic and plasma amino acid responses to equi-carbohydrate milk meals, a pilot- study of bovine and human milk

**DOI:** 10.1186/1475-2891-11-83

**Published:** 2012-10-12

**Authors:** Ulrika Gunnerud, Jens J Holst, Elin Östman, Inger Björck

**Affiliations:** 1 Department of Applied Nutrition and Food Chemistry, Lund University, P.O. Box 124, 221 00, Lund, Sweden; 2 Department of Medical Physiology, The Panum Institute, University of Copenhagen, Copenhagen, Denmark

**Keywords:** Amino acids, Bovine milk, GIP, GLP-1, Human milk, Whey protein

## Abstract

**Background:**

Dairy proteins, in particular the whey fraction, exert insulinogenic properties and facilitate glycemic regulation through a mechanism involving elevation of certain plasma amino acids, and stimulation of incretins. Human milk is rich in whey protein and has not been investigated in this respect.

**Method:**

Nine healthy volunteers were served test meals consisting of human milk, bovine milk, reconstituted bovine whey- or casein protein in random order. All test meals contributed with 25g intrinsic or added lactose, and a white wheat bread (WWB) meal was used as reference, providing 25g starch. Post-prandial levels in plasma of glucose, insulin, incretins and amino acids were investigated at time intervals for up to 2 h.

**Results:**

All test meals elicited lower postprandial blood glucose responses, expressed as iAUC 0–120 min compared with the WWB (P < 0.05). The insulin response was increased following all test meals, although only significantly higher after whey. Plasma amino acids were correlated to insulin and incretin secretion (iAUC 0–60 min) (P ≤ 0.05). The lowered glycemia with the test meals (iAUC 0–90 min) was inversely correlated to GLP-1 (iAUC 0–30 min) (P ≤ 0.05).

**Conclusion:**

This study shows that the glycemic response was significantly lower following all milk/milk protein based test meals, in comparison with WWB. The effect appears to originate from the protein fraction and early phase plasma amino acids and incretins were involved in the insulin secretion**.** Despite its lower protein content, the human milk was a potent GLP-1 secretagogue and showed insulinogenic properties similar to that seen with reconstituted bovine whey-protein, possibly due to the comparatively high proportion of whey in human milk.

## Background

The prevalence of type 2 diabetes (T2D) is increasing around the world, with sedentary lifestyles and inadequate dietary habits as the major causing factors. Epidemiological evidence suggests, that a diet characterized by a low glycemic index (GI), protects against the development of T2D [1]. The GI is used to establish post-meal glycemic responses to carbohydrate rich meals. Most often the GI correlates with Insulinemic Index (II), measured similarly from postprandial insulinemic responses. However, milk displays a discrepancy, showing a low GI but yet a high II [2]. Observational studies have indicated that a diet rich in dairy products may protect against development of the metabolic syndrome and T2D [3,4]. Additionally, bovine whey protein stimulates insulin secretion and facilitates post-meal glycemic regulation in both healthy individuals and in patients with T2D [5-8]. This may imply that co-ingestion of milk, in particular the whey fraction, with meals may lower glycemic excursions and thereby reduce the glycemic impact of the diet.

In a previous study, we have shown that whey protein resulted in a higher insulin response than cheese (casein) or milk (whey and casein), respectively [6]. The major insulin releasing properties of milk could thus be ascribed to the whey fraction, and the mechanism appears to be related to the specific amino acid pattern appearing in postprandial blood after whey ingestion [6]. When administrated with carbohydrates, dietary amino acids stimulate the insulin release from the pancreatic β-cell [9]. Especially leucine (leu) is a known to be strong insulin secretagogue [10]. Consequently, the lowering of glycemia seen when adding whey to a carbohydrate containing meal can to a large extent be mimicked by supplementation of the meal with specific amino acids [11]. In addition, whey proteins appear to stimulate the incretin hormones glucose-dependent insulinotropic polypeptide (GIP) and glucagon-like peptide 1 (GLP-1) [12]. Both GLP-1 as well as GIP has been identified as strong insulinotropic agents [13]. Furthermore, it has recently been reported that dietary branched chain amino acids (BCAA) facilitates GLP-1 release from intestinal-cells [14]. Thus, plasma amino acids per se can possibly cause an incretin secretion, and partly explain the reduction of glycemia seen when adding whey to a carbohydrate containing meal.

The protein concentration in human milk is considerably lower than in bovine milk (8.5 g/L vs. 34.5 g/L) [15]. Also the composition of the protein fractions is substantially different, with human milk containing a larger fraction of whey protein. Consequently, the ratio whey:casein differs between the two milk types, being 20:80 for bovine milk [16] and 50:50 – 80:20 to human milk, with somewhat different ratios depending on the lactating phase [17]. Based on above, it can be hypothesized that human milk, despite its low protein content, exerts insulinogenic properties due to its high whey protein content. To our knowledge, no data are available regarding the postprandial glycemic, insulinemic and incretin effects of human milk.

The aim of the present study was to investigate the glycemic and insulinemic effects of human and bovine milk in comparison with white wheat bread (WWB). In addition, casein and whey protein fractions from bovine milk were included. For this purpose, human milk, bovine milk, bovine casein and whey proteins, respectively, were standardized with respect to lactose content and provided as breakfast drinks to healthy adults. The study was performed in a glycemic index measuring setting, and a carbohydrate equivalent amount of white wheat bread (WWB) was therefore used as reference meal. In addition to blood glucose and insulin, plasma levels of amino acids and the incretins GIP and GLP-1 were measured in the postprandial phase.

## Methods

### Test meals

Four test drinks were included in the study; human and bovine milk and reconstituted bovine whey and casein drinks. The nutritional compositions of the meals are shown in Table 1. Human milk, whey and casein concentrates were obtained from Arla Foods (Stockholm, Sweden). Bovine milk with 1.5% fat (w/v) was obtained from a local market. The human milk was skimmed to reduce fat content (1.5% w/v), followed by pasteurization. According to the manufacture the whey and casein proteins were obtained by ultrafiltration and membrane filtration of skimmed milk. Lactose was then added to the protein fractions and the mixtures were dissolved in water. Each of the meals contributed with 25 g available carbohydrates, thus the protein content differed significantly between the bovine milk and the human milk meals (16.8 g and 3.5 g respectively). The reconstituted whey and casein meals were set to match the bovine milk with respect to lactose-, protein and fat contents. The serving of whey contained 16.2 g of protein and the casein meal contained 16.8 g protein. All the test drinks were set to match the bovine milk in fat content (1.5% w/v). Human milk, whey and casein drinks were kept frozen (−18°C) until use. White wheat bread (WWB) was used as a reference meal. The WWB was prepared in a home baking machine according to Liljeberg and Björck [18].

**Table 1:** Nutrient composition and serving sizes of the test meals

	**WWB**^**1**^	**Whey**^**2**^	**Casein**^**2**^	**Bovine milk**^**2**^	**Human milk**^**2**^
Starch (g)	25	−	−	−	−
Lactose (g)	−	25	25	25	25
Protein (g)	3.7	16.2	16.8	16.8	3.5
Fat (g)	2.5	7.4	7.7	7.6	5.7
Serving quantity (g)	250	490	510	510	379
Σ Carbohydrates (g)	25	25	25	25	25

^1^ Solid food.

^2^ Liquid foods.

### Test subjects and study design

Nine healthy, non-smoking, volunteers, (6M: 3F), aged 22–30 years, with body mass index 25.8 ± 3.4 kg/ m^2^ (mean ± SD) and without drug treatment participated in the study. All subjects had normal fasting blood glucose levels (4.4 ± 0.05 mmol/l). The meals were served in random order as breakfast meals at 5 different occasions, at least 2 days apart. When arriving at the laboratory in the morning (8 am), a peripheral catheter was inserted into the antecubital vein and a fasting blood sample was drawn. At all sampling time points, samples were taken in one tube for serum and one for plasma (EDTA), respectively. The subjects were requested to consume the test and reference meals steadily over a 12 min period. All subjects were conscious of the possibility to withdraw from the study at any time they desired. The study was approved by the Ethics Committee of the Faculty of Medicine at Lund University.

### Chemical analysis

Lactose content of the test meals was analyzed as galactose and glucose following enzymatic hydrolysis with β-galactosidase as described by Nilsson *et al.*[6].The peptide-bound amino acids of the different food proteins were analyzed accordingly Stenberg *et al.*[19] starting with a hydrolysis step. The samples were dissolved in 6 M hydrochloric acid, containing 0.1% phenol and kept at 110°C for 20 h. As a result of the hydrolysis treatment, tryptophan, cysteine and methionine were lost and therefore data for these amino acids are lacking in the results. Furthermore, due to the hydrolysis step, glutamine and asparagine were converted to glutamic acid and aspartic acid, respectively. The amino acids were analyzed using ion exchange chromatography (Biotronik LC 5001, München, Germany). Standard lithium citrate buffers of pH 2.85, 2.89, 3.20, 4.02 and 3.49 were used to separate the amino acids. The post-column derivatization was performed with ninhydrin [20]. The crude protein content in all the meals was analyzed by using the Kjeldahl procedure (Kjeltec Auto 1030 Analyser; Tecator Höganäs, Sweden). The starch in the WWB was determined according to the method of Holm et al. [21].

### Blood analysis

Venous blood samples were taken at fasting (0) and, at 7.5, 15, 30, 45, 60, 75, 90, 105 and 120 min after the meal were initiated, for analysis of blood glucose, serum insulin and GIP and GLP-1 analysis. Additionally, plasma was collected at 0, 7.5, 15, 30, 45 and 60 min for amino acids analysis. The plasma tubes were allowed to rest for 30 min before being centrifuged at 3000 rpm during 6 min. 800 μl plasma was separated for free amino acids analysis and the remaining plasma was frozen at −20°C before analysis of GIP and GLP-1. Serum tubes were centrifuged for 15 min at 4000 rpm and serum was separated and frozen at −20°C for insulin analysis. Whole blood glucose concentrations were determined with a glucose oxidase-peroxidase reagent. The serum insulin determination was performed on an integrated immunoassay analyzer, CODA™ Open Microplate System (Bio-Rad Laboratories, Hercules, USA) using an enzyme immunoassay kit (Mercodia Insulin Elisa, Mercodia AB, Uppsala, Sweden). Plasma GIP and GLP-1 concentrations were measured after extraction of plasma with 70% ethanol (by vol, final concentration). For the GIP radioimmunoassay [22], we used the C-terminal directed antiserum R 65, that cross-reacts fully with human GIP but not with so called GIP 8000, whose chemical nature and relation to GIP secretion is uncertain. Human GIP and ^125^I human GIP (70 MBq/nmol) were used for standards and tracers. The concentration of plasma GLP-1 was measured [23] against standards of synthetic GLP-1 7–36 amide by using antiserum code no. 89390, which is specific for the amidated carboxyl terminus of GLP-1 and, therefore, does not react with GLP-1 containing peptides from the pancreas. For both assays sensitivity was <1 pmol/L, intraassay CV <6% at 20 pmol/L, and recovery of standard, added to plasma before extraction, ≈100% when corrected for losses inherent in the plasma extraction procedure. Free amino acids were purified by mixing 200 μl 10% sulphosalic acid with 800 μl plasma to precipitate high molecular proteins, according to Biotronik (München, Germany). The amino acid solutions were frozen at −20°C until analyzed by an amino acid analyzer (Biotronik LC 5001, München, Germany) using ion exchange chromatography as described above.

### Calculations and statistical methods

The results are expressed as mean ± SEM. The incremental areas under the curves (iAUC) for glucose, insulin, GIP, GLP-1 and plasma amino acids were calculated for each test subject and meal, using a trapezoid model (GraphPad Prism™, version 4.03). All areas below the baseline were excluded from the calculations and each subject was their own reference. Statistical comparisons for iAUC-values were performed in MINITAB® Statistical Software (release 14.13 for windows). Significances were evaluated with the general linear model (ANOVA), followed by Tukey’s multiple comparisons test. Statistical significances were considered for values of P < 0.05. Differences between the products at different time points were analyzed by using a mixed model (PROC MIXED in SAS release 9.1; SAS Institute Inc, Cary, NC) with repeated measures and an autoregressive covariance structure. When significant interactions between treatment and time were found, Tukey’s multiple comparison test were performed for each time point (MINITAB, release 14.13; Minitab Inc). Correlations between dependent measures were conducted using Spearman's partial correlation coefficients controlling for subjects and corresponding baseline values (two-tailed test), (SPSS software, version 19; SPSS Inc., Chicago, IL, USA). Due to missing values the bovine milk had to be withdrawn from the GLP-1 and GIP statistical calculations, and the amino acid isoleucine (ile) had to be excluded from the calculations on free amino acids in plasma. In addition, there were only eight test subjects on the WWB and whey, and seven test subjects on the casein and the human milk respectively included in the GIP and GLP-1 calculations, due to missing values.

## Results

### Postprandial blood glucose and insulin responses

**Figure 1:**
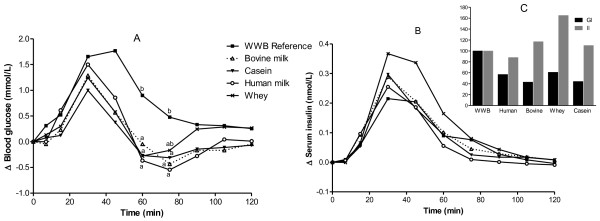
**Incremental changes in plasma glucose and serum insulin.** Mean incremental changes (Δ) in plasma glucose (**A**) and serum insulin (**B**) in response to equal amounts of carbohydrate. In (**C**) the GI and II (iAUC) are displayed. For plasma glucose a significant treatment effect (p < 0.0002) and time × treatment interaction (p < 0.0265) were found at a given time. Values with different lowercase letters are significantly different, p ≤ 0.05 (Tukey’s test). n = 9 healthy subject.

The iAUCs for plasma glucose and serum insulin are shown in Table 2. All test meals elicited lower postprandial blood glucose responses, expressed as iAUC 0–120 min, compared with the reference (WWB) (p < 0.05). A significant treatment effect (p = 0.0002) and a significant time × treatment interaction (p = 0.0265) was found for blood glucose concentrations (Figure 1). The insulin iAUC following human milk, bovine milk and casein was similar to that seen with WWB. In contrast, the whey meal exhibited higher insulin iAUC 0–120 min, than the WWB (p < 0.05). Additionally, the whey meal resulted in an elevated insulin response (iAUC 0–120 min) also when compared with data for casein and human milk. No time × treatment interaction was found for insulin responses (Figure 1).

**Table 2:** Postprandial incremental areas under the curve (iAUC 0–120 min) for plasma glucose, serum insulin, plasma GLP-1 and GIP

	**Glucose**^**1**^		**Insulin**^**1**^		**GLP-1**^**2**^		**GIP**^**2**^	
	**mmol · min**	**%**^**4**^	**nmol · min**	**%**^4^	**pmol · min**	**%**^**4**^	**pmol · min**	**%**^**4**^
WWB	96.8 ± 13.6^a^	0	10.1 ± 1.8^a^	0	276.6 ± 118.6^a^	0	1115.2 ± 271.5^a^	0
Human milk	48.0 ± 7.6^b^	−50	9.0 ± 1.6^a^	−10	427.2 ± 131.9^a^	+154	699.6 ± 151.1^a^	−37
Whey	54.1 ± 11.7^b^	−44	16.0 ± 2.6^b^	+58	980.2 ± 167.8^b^	+354	2717.3 ± 665.3^b^	+244
Casein	35.1 ± 8.4^b^	−64	10.4 ± 1.8^a^	+3	444.9 ± 118.8^a^	+180	1260.1 ± 259.2^a^	+113
Bovine milk	40.6 ± 10.9^b^	−58	11.7 ± 2.6^ab^	+16	−		−	

Values are means ± SEM. Values within the same column not sharing same letters are significantly different (P < 0.05).

^1^n = 9 healthy subjects.

^2^n = 8 healthy subjects (WWB, whey) and n = 7 (casein, human milk).

^4^ Change in postprandial response as a percentage of the WWB reference meal.

### Amino acid content in the test and reference meals

The amino acid concentrations of the different meals are presented in Table 3. Bovine milk, casein and whey have similar patterns, with higher concentrations of the BCAA; ile, leu, and valine (val) compared to human milk and WWB. The whey meal stands out by higher leu content, and bovine milk and casein, respectively, have slightly higher amounts of val. In accordance to its lower protein content, the human milk display consistently lower content of all analyzed amino acids compared to the whey, casein and bovine milk. In comparison to the WWB, the human milk has higher concentrations of val, leu and lys, whereas the WWB have high proline, glycine and tyrosine (tyr) levels.

**Table 3:** Content of amino acids in the different meals

	**Meal**				
**Amino acid**	**WWB**	**Whey**	**Casein**	**Bovine milk**	**Human milk**
**mg/serving**					
Ala	144	760	449	500	125
Arg	80	387	525	525	129
Asp	55	1539	969	1097	273
Glu	149	2592	3341	3529	678
Gly	509	314	296	321	117
His	87	284	413	423	83
Ile	163	921	872	770	136
Leu	134	2117	1698	1668	368
Lys	191	1490	1214	1306	243
Phe	96	529	745	765	133
Pro	1344	760	1698	1734	337
Ser	98	573	765	796	136
Thr	144	715	638	689	152
Tyr	293	564	847	699	133
Val	111	760	974	1000	197

### Postprandial plasma amino acids

The 0–60 min iAUC for plasma amino acids are displayed in Table 4. The postprandial responses for val, leu, lys, serine (ser), alanine and arginine (arg) were significantly higher following whey, casein and bovine milk compared to the WWB. Furthermore, casein and whey resulted in a significantly higher iAUC for arg and ser compared to the human milk. The tyr response was significantly higher after the whey protein and the bovine milk, respectively, compared with the WWB and the human milk. The whey and casein meals also elicited significantly higher plasma iAUC for threonine than all the other meals. There were no differences between the human milk and the WWB in any of the analyzed amino acids in postprandial plasma.

**Table 4:** Postprandial areas under the curve (iAUC 0–60 min) for the different plasma amino acids

	**Meal**				
**Amino acid**	**WWB**	**Whey**	**Casein**	**Bovine milk**	**Human milk**
**(mmol · min/L)**					
Ala	0.4 ± 0.2^a^	3.5 ± 0.7^b^	2.4 ± 0.6^b^	2.7 ± 0.6^b^	2.1 ± 0.5^ab^
Arg	0.2 ± 0.1^a^	1.0 ± 0.2^b^	0.9 ± 0.2^b^	0.9 ± 0.2^b^	0.5 ± 0.1^ab^
Glu	0.9 ± 0.5^a^	3.0 ± 1.1^a^	2.9 ± 0.5^a^	1.7 ± 0.5^a^	1.3 ± 0.5^a^
Gly	0.3 ± 0.1^a^	0.5 ± 0.1^a^	0.5 ± 0.2^a^	0.4 ± 0.2^a^	0.2 ± 0.1^a^
Hist	0.3 ± 0.1^a^	0.6 ± 0.1^a^	0.4 ± 0.1^a^	0.6 ± 0.2^a^	0.7 ± 0.4^a^
Leu	0.3 ± 0.1^a^	5.0 ± 0.5^c^	2.7 ± 0.5^b^	2.6 ± 0.5^b^	0.6 ± 0.2^a^
Lys	0.5 ± 0.3^a^	4.7 ± 0.6^b^	2.9 ± 0.6^b^	2.9 ± 0.6^b^	1.0 ± 0.3^ab^
Phe	0.5 ± 0.2^a^	0.5 ± 0.2^a^	0.5 ± 0.1^a^	0.3 ± 0.1^a^	0.3 ± 0.1^a^
Pro	2.4 ± 0.6^a^	2.4 ± 0.7^a^	2.1 ± 0.3^a^	3.0 ± 0.5^a^	1.5 ± 0.4^a^
Ser	0.1 ± 0.1^a^	1.2 ± 0.2^c^	0.9 ± 0.2^c^	0.8 ± 0.2^bc^	0.3 ± 0.1^ab^
Thr	0.2 ± 0.1^a^	1.7 ± 0.3^b^	1.3 ± 0.1^b^	0.8 ± 0.2^a^	0.6 ± 0.2^a^
Tyr	0.2 ± 0.1^ab^	1.0 ± 0.3^b^	0.6 ± 0.2^ab^	1.1 ± 0.4^b^	0.1 ± 0.1^a^
Val	0.6 ± 0.2^a^	3.3 ± 0.5^b^	3.0 ± 0.4^b^	2.3 ± 0.5^b^	0.6 ± 0.2^a^

Values are means ± SEM; n = 9. Values in the same column with different letters are significantly different, p < 0.05 (ANOVA followed by Tukey’s test).

### Postprandial GLP-1 and GIP responses

**Figure 2:**
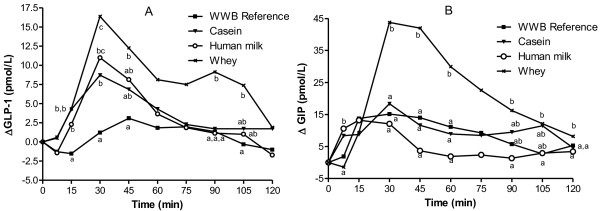
**Incremental changes in plasma GIP and GLP-1.** Mean incremental changes (Δ) in plasma GLP-1 (**A**) and GIP (**B**) in response to equal amounts of carbohydrate. Significant treatment effects (p < 0.001) as well as time × treatment interactions (p < 0.001) were found for both GLP-1 and GIP. Values with different letters are significantly different, p < 0.05 (Tukey’s test). n = 8 (WWB, whey) and n = 7 (human milk, casein).

The iAUCs 0–120 min for GLP-1 and GIP are shown in Table 2. The whey meal resulted in a significantly higher iAUC 0–120 min for both GIP as well as GLP-1, in comparison to all the other meals. Also in the case of the iAUCs 0–45, 0–60 and 0–90 min, respectively the whey meal elicited higher increment for both GIP and GLP-1 compared with WWB (p ≤ 0.05). In addition, the human milk elicited a significantly higher early postprandial GLP-1 response, expressed as iAUCs 0–30 and 0–45 min, compared to the WWB (p ≤ 0.05) (early iAUC not displayed). Incretin responses following the casein meal showed no differences compared to the WWB. Significant differences between treatments over the entire time course (p ≤ 0.001) and significant time × treatment interactions (p ≤ 0.001) were found for both GLP-1 and GIP responses (Figure 2).

### Correlations between parameters

Correlations between plasma amino acids, serum insulin and plasma incretins are displayed in Table 5. Positive correlations were observed between the plasma amino acids leu, lys, val and thr iAUC 0–60 min and the corresponding iAUC for serum insulin, and plasma incretin responses. The individual plasma amino acids (leu, lys, val and thr) iAUC 0–45 min correlated inversely to glucose response at iAUC 0-60min (data not showed). In addition, early phase GLP-1 response, expressed as iAUC 0–30 min, correlated positively to the early phase insulin response (iAUC 0–30 min; r = 0.688, p < 0.001). Moreover, did the early phase GIP and GLP-1, expressed as iAUCs 0–15 min, correlate negatively with blood glucose responses iAUC 0–90 min (r = −0.419, p = 0.040; r = −0.374, p = 0.012, respectively).

**Table 5:** Correlations between serum insulin, GIP and GLP-1 and plasma amino acids

		**Insulin**	**GLP-1**	**GIP**
Leu	r	0.440	0.716	0.489
	p	0.003	<0.001	0.007
Lys	r	0.385	0.640	0.568
	p	0.010	<0.001	<0.001
Thr	r	0.364	0.600	0.383
	p	0.015	<0.001	0.040
Val	r	0.408	0.438	0.539
	p	0.006	0.017	0.003

Spearman’s correlation coefficients and *p-*values for the relations between increments in plasma amino acid concentrations and the corresponding serum insulin, plasma GLP-1 and GIP increment, respectively (iAUC 0–60 min).

## Discussion

To the best of our knowledge, this is the first study where human milk is evaluated with respect to glycemic and insulinemic effects. The study was performed in healthy adults with a GI setting, thus post prandial glycemic and hormonal responses to the human and bovine milk were compared to an equicarbohydrate amount of WWB. In the present study, we found that human milk displayed insulinogenic properties and resulted in a postprandial glycemic response (GI 57) that was in the same range as that to bovine milk (GI 43) or the whey and casein fractions, respectively (GI 61 and 44). The human milk showed the lowest insulin response (iAUC 0–120 min) in comparison to all the other meals, thus indicating that human milk display superior insulin economy. Worth emphasizing is that the protein content of the human milk meal was markedly lower than that of bovine milk, whey and casein meals. According to our analysis, the human milk meal contained 3.5g protein as calculated from nitrogen analysis using 6.38 as conversion factor. However, it has been has reported that the true protein content of human milk is overestimated from nitrogen analysis due to its high non protein nitrogen (NPN) content (20-25% of total nitrogen) [16,17]. Consequently, the protein amount in the human milk, used in this study, may have been overestimated. Although the whey:casein ratio of the human and bovine milk in this study was not analyzed, it has been reported to range from 50:50 – 80:20 in human milk depending on the lactation period [17], compared to approximately 20:80 in commercial bovine milk [16]. Our results thus indicate that human milk may be more insulinogenic per unit protein compared to bovine milk, and the higher proportion of whey protein may be a contributing factor.

All the test products in our study yielded higher postprandial plasma amino acid responses compared to the WWB reference meal, although not reaching significance for all products. The bovine milk and the reconstituted whey and casein meals were especially prone to affect responses of the BCAA; val and leu, and also lys and thr. We also noticed a tendency to a rise in these amino acids following human milk in comparison to the WWB. We recently showed *in vitro* that the amino acids ile, leu, val, lys and thr induce strong insulin secreting properties in mouse pancreatic islets [24]. In particular, leu is recognized as a potent insulin secretagogue [25,26]. Moreover it has been reported that also taurine, the second most common “amino acid” and the largest part of the NPN fraction in human milk [27,28], may be involved in insulin secretion [29,30]. In the present study, we also observed positive correlations between individual plasma amino acids and serum insulin and plasma incretin secretion in the postprandial phase (iAUC 0–60 min) as well as negative correlations to the glycemic response (iAUC 0-60min). Altogether this suggests that the amino acids play an important role in the insulinogenic properties of the milk proteins and contribute to lowered postprandial glycemia. Thus, it can be suggested that amino acids appearing in plasma following milk ingestion, may affect insulin secretion in two ways; directly by acting on the pancreatic β-cells and indirectly by promoting incretin release. We thus found that human milk, bovine whey and casein respectively, affect release of GLP-1, with the human milk resulting in a significantly higher early response compared to the WWB meal. In relation to the human milk, the whey meal yielded a higher GLP-1 response. However, considering the fact that the protein content in the human milk meal constitutes only about 22% of that in the bovine whey meal, the early GLP-1 response (iAUC 0–30 min) still reached more than 50% of that seen following whey. Thus, it could be suggested that human milk is prone to stimulate GLP-1. It remains to be shown if the GLP-1 response is solely related to the proteins in the human milk, or if other bioactive components may also be involved.

Strong positive correlations were found between the early GLP-1 response (iAUC 0–15 min) and postprandial insulin release up to 30 min, as well as negative correlations to blood glucose response (iAUC 0–90 min), an indication that early GLP-1 release may be important for the modulation of glycemia following both human and bovine milk. In support of such an opinion, we recently observed that ingestion of whey protein and free amino acid caused an early GLP-1 secretion that reduced postprandial glycemia in absence of a hyper-insulinemic peak [31]. In addition to GLP-1, the human milk also resulted in a small elevation of early phase GIP that was significantly higher than after the WWB reference meal at 7.5 min. GIP may be present in human milk [32], and the response we found might result from a higher intake of this incretin, and could possibly attenuate the postprandial insulin response and facilitate glycemic regulation. Although, little is known about to what extent ingested GIP can maintain activity following exposure to the acidic environment in the stomach, it could be hypothesized that ingestion of intrinsic GIP could possibly contribute to the incretin effect of human milk. In previous studies of incretin responses we have preferentially seen increase in GIP after whey ingestion [5]. Thus, it is interesting that the whey meal, as well as the human milk meal, in this study significantly increased both GLP-1 and GIP response compared to all the other meals. GLP-1 secretion following whey ingestion has previously been reported by Hall et al. [33]. However, in that study the test meals contained 38E% of fat, which may have influenced GLP-1 secretion. Both GLP-1 and GIP have been identified as strong insulinotropic agents [13,34], and a release of both these incretins after whey ingestion may add to its insulinogenic properties. Interestingly, it was recently reported that whey protein inhibits dipeptidyl peptidase IV (DPP-4), an enzyme that inactivates GLP-1 [35]. Consequently, whey protein may both induce GLP-1 secretion as well as prolong the activity of this incretin.

In contrast to whey, the casein meal resulted in a small incremental insulin response and had the lowest glycemic response compared to the other test meals. This differences in glycemic and insulinemic responses when, comparing the casein and whey meal, is in contrast with observations by Hall et al. [33], who found no differences on neither postprandial glucose nor insulin responses between these proteins. However, in accordance with the present study, they observed that whey protein, in comparison to casein, increased plasma incretins, with higher levels of both GLP-1 and GIP [33].

There were some limitations of the present study. All the test meals resulted in lower blood glucose increments compared to the WWB reference meal. It should, however, be noted that lactose was the carbohydrate source in all the test meals, whereas the reference WWB meal contained starch. In previous studies, we have shown that lactose has a lower glycemic index (GI 68) and insulinemic index (II 50) than the starchy WWB (GI 100 and II 100) [2]. Consequently, the comparatively low glycemic properties seen with all the present test meals could partly be explained by the lactose per se. The number of test subject is slightly lower (n = 9) than recommended for standardized glycemic index determinations (n = 10) [36]. Also, although the test subjects were used to regular Swedish food habits where milk consumption is frequent, differences in lactase levels might affect glycemia to milk.

## Conclusion

The present study showed that human milk as well as bovine milk and whey and casein protein lowered the glycemia compared to the WWB reference meal. We also demonstrated that the human milk exerted insulinogenic properties and the effect appeared to originate from the protein fraction. Consequently, all the test meals increased plasma amino acid levels, and the amino acids correlated positively with both insulin secretion and incretin responses as well as inversely to the glycemic response. Interestingly, the human milk appeared to be a particularly potent GLP-1 secretagogue. The relative potency of human milk to stimulate incretins is noteworthy and the impact of incretins for metabolism and appetite regulation is an interesting field for further research and should be taken into consideration when producing baby formulas based on bovine milk.

## Abbreviations

iAUC: Incremental area under the curve; BCAA: Branched chain amino acids; GI: Glycemic index; GIP: Glucose-dependent insulinotropic polypeptide; GLP-1: Glucagon-like peptide 1; II: Insulinemic index; NPN: Non-protein nitrogen; T2D: Type 2 diabetes; WWB: White wheat bread.

## Competing interests

The authors declare that they have no competing interest.

## Authors’ contributions

UG was responsible for sample collection and analysis of the data, statistical analysis and for writing the paper. JJH was responsible for the incretin analysis. EÖ was involved in interpretation of data and in writing the paper. IB was responsible for the study design and coordination of the study, securing the funding and was involved in the evaluation and in writing the paper. All authors read and approved the final manuscript.
